# The Impact of Brewer's Spent Grain on Noodle Quality and Composition

**DOI:** 10.1111/1750-3841.71428

**Published:** 2026-08-31

**Authors:** Gabriely M. Soncin Alfaro, Shaun J. Clare, Robert S. Brueggeman, Sean M. Finnie, Alecia M. Kiszonas

**Affiliations:** ^1^ School of Food Science Washington State University Pullman Washington USA; ^2^ Department of Crop and Soil Sciences Washington State University Pullman Washington USA; ^3^ United States Department of Agriculture Agriculture Research Service, Western Wheat Quality Laboratory Pullman Washington USA

## Abstract

Brewer's spent grain is a large, highly nutritious fraction remaining after the brewing of beer. Spent grain is usually used as animal feed, when destinations are closely available. It is also discarded in landfills, leading to economic and environmental concerns. This study explored the use of spent grain to enhance the nutritional content of fresh yellow alkaline noodles while also decreasing spent grain waste. Spent grain from a malting barley variety was produced, oven‐dried, milled, and blended with wheat flour at 5%, 15%, and 25% of spent grain (w/w) to produce noodles. The impact of these test formulations on noodle quality, as well as composition, measured as total arabinoxylans and β‐glucans, was investigated for both raw and cooked noodles. A commercial sample of buckwheat noodles (BW) was also tested for comparison. On average, noodles with 25% spent grain cooked 2 min faster than BW and a wheat control made without the addition of spent grain (*p* < 0.05). Spent grain noodles were slightly firmer, had lower cooking loss, and had lower weight increase compared to the control noodles. Portions of 100 g of cooked noodles made with 25% spent grain had 2.2 g more fiber than control noodles, measured as total arabinoxylans and β‐glucans. The inclusion of 25% spent grain is a viable way to enhance noodle fiber content while maintaining noodle quality.

**Practical Applications:**

Dried and ground spent grain can be incorporated into noodles, simultaneously improving noodle fiber content and reducing cooking time. Noodles represent a potential new market for spent grain, preventing byproduct waste.

## 1 Introduction

Brewer's spent grain, also known as barley spent grain or spent grain, is a byproduct of the brewing industry, being the remaining solid fraction after the mashing step in the beer‐making process. Spent grain consists of the husk, pericarp, and seed coat fractions of the barley grain (Mussatto et al. 2006; Steiner et al. 2015). Spent grain accounts for 85% of all the residue generated during beer manufacturing (Mussatto 2014; Steiner et al. 2015). The production of 100 L of beer generates approximately 20 kg of spent grain (Mussatto et al. 2006). In 2024, an estimate of 178 billion liters of beer were produced globally, and beer production is projected to stay steady for the next 5 years (Statista Consumer Markets Insights 2025). With such a high production volume, there is great opportunity for exploring the use of spent grain.

Approximately one‐third of spent grain is used for animal feed, which must be distributed locally due to the high moisture content of this fresh residue; however, much of the remaining spent grain is discarded in landfills, contributing to greenhouse gas emissions (Mussatto et al. 2006; Lamas and Gende 2023; Pérez‐Alva et al. 2025).

Spent grain contains minerals, B‐vitamins, essential amino acids (Huige 2006), and antioxidant compounds including phenolic acids and flavonoids (Pérez‐Alva et al. 2025). A highly nutritious fraction due to its high protein and fiber content, spent grain can supplement human nutrition (Steiner et al. 2015; Lynch et al. 2016; Shi et al. 2023; Ahuja et al. 2024). The composition of spent grain varies considerably based on different raw materials and brewing conditions (Lynch et al. 2016). Typical levels of protein range from 19% to 30% and fiber levels can vary from 30% to 50% (Lynch et al. 2016). Arabinoxylans and mixed linkage (1→3, 1→4)‐β‐D‐glucans (β‐glucans) are the predominant non‐starch polysaccharides present in barley (Izydorczyk and Dexter 2008) and, consequently, are present in spent grain (Steiner et al. 2015). The health benefits associated with the consumption of dietary fiber in the form of arabinoxylans and β‐glucans include lowering postprandial glycemic responses and reducing blood cholesterol, thereby reducing the risk of heart disease (Steiner et al. 2015; Lynch et al. 2016).

The incorporation of spent grain into food products brings environmental and nutritional advantages (Steiner et al. 2015). The inclusion of spent grain to fortify wheat‐based food products for nutritional benefits has been previously reported. A blend of soft wheat flour and 10% of dried, sifted spent grain increased cookie dietary fiber content from 1.5% to 3.5%, and resulted in cookies with acceptable diameter and appearance; inclusion levels up to 40% of spent grain elevated the protein content to 11% and the fiber content to 9.1% but required the addition of soy lecithin (Kissell and Prentice 1979). In baked snacks, the inclusion of 25% spent grain increased the total fiber content of the product from 6.3% to 20.1%, but changed the odor profile and negatively affected the crumb structure and texture of the samples (Ktenioudaki et al. 2013). However, at a lower inclusion level of 10% spent grain, the crispiness of the snacks was not affected, and the product was sensorially acceptable, with a total fiber content of 11.9% (Ktenioudaki et al. 2013). The incorporation of 6% spent grain significantly increased the fiber content of bread made with refined and whole wheat flour from 1.5% to 3.1% (*p* < 0.05) (Lamas and Gende 2023). Another study investigated bread formulations with 0%, 10%, 20%, and 30% spent grain in bread (Stojceska and Ainsworth 2008). Inclusion of spent grain increased the total dietary fiber content of the studied breads from 2.3% in the control bread without spent grain to up to 11.5% in the bread with 30% spent grain (Stojceska and Ainsworth 2008). Although no differences were observed in the crumb structure, adding 20% or 30% spent grain increased crumb firmness and reduced loaf volume, which could be alleviated using appropriate enzymes (Stojceska and Ainsworth 2008).

A survey conducted to investigate consumer attitudes towards upcycled foods showed that spent grain was the top byproduct category selected as suitable to be upcycled, chosen by 19% of the US participants (Grasso et al. 2023). In an extensive list of food categories, “pastas” including dry pasta and noodles, was on the top three most selected food by the US participants when asked “In which new foods (if any) would you like to see the upcycled ingredients to be re‐introduced?,” behind the most selected categories of “snacks” and “breakfast” (Grasso et al. 2023). The willingness to purchase upcycled foods was recently reported for a broad range of age groups: Gen Z (born between 1995 and 2015), Gen Y (1980–1994), and Baby Boomers (1944–1964) (Zhang et al. 2021), which could be potential consumers for products with spent grain.

Noodles are a convenient, popular food, and are consumed globally (World Instant Noodles Association 2025). The nutritional enhancement of noodles through the incorporation of food byproducts has been explored. A previous study concluded that substituting wheat flour with up to 11% of dragon fruit peel increased the protein content of wet noodles from 6.6% to 7.9%, whereas the incorporation of soybean curd residue reduced noodle protein content to 5% (Manurung et al. 2024). In another study, the use of apricot kernel flour to substitute 15% of the wheat flour in the formulation of dried noodles increased the protein content of the product from 11.5% to 13.5% and resulted in noodles with acceptable sensory and physicochemical properties (Eyidemir and Hayta 2009). Replacing wheat flour with watermelon rind powder at substitution levels of 5%, 10%, and 15% decreased the protein content while increasing the dietary fiber levels of wet noodles from 0.1% in the control noodles to 2.4% for noodles at the highest substitution level (Ho and Che Dahri 2016).

Soba noodles have brown or grey color (Fu 2008) and are popular in Japan and Korea (Hou et al. 2010). Inclusion levels of 30%–40% buckwheat (BW) (*Fagopyrum esculentum*) flour can be used in the formulation of soba noodles (Hou et al. 2010). However, Asian countries have a high incidence of individuals allergic to BW, especially children (Kajita and Yoshida 2024). The exposure to BW can cause severe reactions in sensitive individuals, including anaphylaxis (Kajita and Yoshida 2024).

To investigate a new food application for a brewing byproduct, this study explored the suitability of the use of oven‐dried spent grain and wheat flour as blends to produce yellow alkaline noodles. Pre‐tests suggested the adequate levels of spent grain inclusion. Three levels of inclusion were tested (5%, 15%, and 25% spent grain) for their impact on noodle quality parameters, including texture and color. Anticipated benefits included the enhancement of noodles by the inclusion of a fiber‐rich ingredient and the repurposing of a byproduct that currently goes to waste. Because of the visual similarities in the color and the specks of noodles with spent grain and BW noodles, this study also compared the quality parameters of the developed noodles and a commercial sample of BW noodles.

## 2 Materials and Methods

### 2.1 Plant Material

This study created noodles from a composite of flour milled from Keldin wheat and inclusions of spent barley grain created as a byproduct of the malting process. The barley accession 20WAM‐783.1 used in this study was developed as a malting variety. The barley seeds were grown and harvested under field conditions at Spillman Farm, Pullman, WA, in 2023. Keldin (PI 669490) is a popular Pacific Northwest (PNW) hard red winter (HRW) wheat variety released by WestBred (Bayer AG) in 2014. The wheat samples were harvested across different locations in the PNW in 2023 and milled according to Jeffers and Rubenthaler (1977) (USDA‐ARS Western Wheat Quality Lab [WWQL], Pullman, WA, USA).

### 2.2 Creation of Spent Grain

#### 2.2.1 Malt Parameters

A total of 4.5 kg of 20WAM‐783.1 seed was malted in a Curio Micromalting system (Milton Keynes, UK). First, grain was steeped in 14°C water for 8 h, followed by a 16 h air rest at 14°C and a second steep in 14°C water for 8 h. Grain was subsequently allowed to germinate for 96 h at 15°C with 100% relative humidity. Green malt was transferred to the kiln and dried at 50°C for 5 h, followed by a drying sequence of 55°C for 5 h, 60°C for 3 h, 72°C for 6 h, and 82°C for 3 h. The resulting malt was transferred to a deculmer to remove rootlets for the final malt.

#### 2.2.2 Spent Grain

The malt was subjected to a standard mash profile. A total of 3.2 kg was placed in a malt pipe and mashed at 105°C for 1 h with distilled water in a DigiMash Electric All Grain 35L Brewing System (Melbourne, Australia). The spent grain was removed from the combined mash tun/kettle with the malt pipe and allowed to drain. The spent grain was then pressed to remove excess water, spread onto an aluminum baking pan, and dried in a convection oven at 100°C for approximately 90 min. Samples of spent grain were taken during drying to measure moisture content with a halogen moisture analyzer (HB43‐S, Mettler Toledo, Greifensee, Switzerland) set to a barley calibration. Drying time was determined as the time required to reach ∼5% moisture content. Dried spent grain was kept in air‐tight containers until milled.

### 2.3 Flour and Flour Blend Parameters

Dried spent grain was milled using an ultracentrifugal mill (ZM 300, Retsch GmbH, Haan, Germany) equipped with a 24‐teeth rotor with speed of 5534 × *g* and a 500 µm sieve. The mill temperature was kept below 24°C and the feed rate was approximately 5.3 g/s. A vibratory sieve shaker (AS 200 Control, Retsch GmbH) was used to sift the product through a 500 and 210 µm sieve. The fraction with particle size larger than 210 µm was passed through the mill a second time set to the same settings as the initial pass. The re‐milled fraction was sifted under the same conditions as the first sifting. The fractions with particle size smaller than 210 µm were combined and used as spent grain flour.

Three test flour blends were created by mixing 5%, 15%, and 25% spent grain with 95%, 85%, and 75% wheat flour (w/w), respectively (Table 1). The composition of wheat flour, spent grain, and flour blends was investigated. Protein content was measured with a nitrogen and protein analyzer (Leco FP828p, Leco Corp., St. Joseph, MI, USA) according to the Approved Method 46‐30.01 of the American Association of Cereal Chemists International (AACCI). Ash and moisture contents were measured with a Leco TGA701 thermogravimetric analyzer. β‐Glucan content was assessed with a Megazyme β‐glucan (Mixed Linkage) assay kit (K‐BGLU) (Megazyme Ltd., Bray, Ireland), following the procedures provided by the manufacturer and according to AACCI Approved Method 32‐23.01. Total arabinoxylans (TAX) were determined according to the method reported by Hernández‐Espinosa et al. (2022) for analysis of total pentosan content in whole meal. The color of the flour and the flour blends was measured with a colorimeter (Minolta CR‐410, Konica Minolta, Osaka, Japan) equipped with a pulsed xenon lamp as the light source and a 2° standard observer. Color measurements were reported as values of L*, a*, and b*.

**TABLE 1:** Description of flour blends and noodle dough hydration.

Sample	Spent grain (%)	Wheat flour (%)	Noodle dough hydration (%)
Control	0	100	33
5	5	95	34
15	15	85	35
25	25	75	36
100	100	0	^a^

*Note*: Spent grain and wheat flour amounts are expressed in percentage of weight by weight (w/w).

^a^ Noodles with 100% spent grain were not produced.

### 2.4 Preparation of Noodles

Noodles were prepared with HRW wheat flour and the flour blends according to the instructions of Hou (2010) for raw yellow alkaline (chuka‐men) noodles. A sample of 100 g of flour or flour blend was used to create each batch of noodles. Sodium chloride (NaCl, 1.0%), potassium carbonate (K_2_CO_3_, 0.6%), and sodium carbonate (Na_2_CO_3_, 0.4%) were pre‐dissolved in deionized (DI) water and added to the flour to make dough. The amount of water added to each flour blend to achieve optimal hydration varied from 33 to 36 mL and was different for each blend (Table 1).

The ingredients were mixed in a standard orbital pin mixer (National Manufacturing Co., TMCO, Inc., Lincoln, NE, USA) for 4 min and 15 s, with a pause halfway through to fold in any dough that adhered to the surface of the bowl. The crumbly dough rested for 5 min in a plastic bag at ambient temperature and was then pressed through the rolls of a noodle machine (Ohtake Noodle Machine Manufacturing Co., Tokyo, Japan). The resulting noodle sheet was compressed to a final thickness of 1.4 ± 0.03 mm and then cut into noodle strands of 1.5 mm width with a #20 square type cutter attached to the noodle machine. These prepared fresh noodles were kept in plastic bags until noodle quality testing. Six batches of each noodle formulation were prepared in total: three batches were used for noodle quality tests, and three batches used to assess noodle composition.

A commercial BW noodle sample (ingredients: wheat flour, BW flour, water, salt) was used in this study as a reference point to compare parameters of noodle quality among the samples created.

### 2.5 Noodle Quality

Cooking time was measured according to AACCI Method 66.51‐01 and reported as the mean of three replicates. For each replicate, a sample of 30 g of noodle was cooked in 400 mL of boiling DI water inside titanium pots (Evernew Co., Tokyo, Japan) to determine cooking time. New 30 g samples were cooked for the fixed cooking time. Cooked noodles were submerged in 200 mL of DI water at 24°C for 30 s, drained, resubmerged in 200 mL of fresh DI water at 3°C for 30 s, and drained again, as an adaptation of AACCI Method 66‐60.01. Noodle weight was recorded before and after cooking to determine weight increase. The cooking and cooling water were collected in a pre‐weighed aluminum can to determine cooking loss. The collected water was dried at 100°C for a minimum of 20 h and the cans were reweighed, following AACCI Method 66‐50.01.

Noodle firmness was tested with a texture analyzer (TA‐XT2i, Texture Technologies, Hamilton, MA, USA) equipped with a 5 kg load cell, following AACCI Method 66‐50.01 and Hou (2010). Five cooked noodle strands were aligned on the equipment platform and firmness was measured with a perpendicular plastic cutting blade (part no. TA‐47). Noodle firmness was recorded on the Texture Exponent 32 software (Stable Micro Systems) with the following settings: pre‐test speed: 4 mm/s; test speed and post‐test speed: 1 mm/s; strain: 70%; time: 1 s; trigger type: auto (force); and trigger force: 10 g. Noodle color was recorded before and after cooking on noodle strands aligned against a black surface. Values of L*, a*, and b* were recorded with a colorimeter (Minolta CR‐410).

### 2.6 Noodle Composition

The compositions of raw (uncooked) and cooked noodles were investigated. Cooked noodles were spread onto a pasta drying rack to dry overnight at room temperature before further processing. Noodles were processed into powder form (particle size < 0.5 mm) on the same equipment and settings described in the Section 2.3, with only one milling pass. Moisture, ash, β‐glucan, and TAX contents of pulverized noodles were measured following the same procedures previously described in the Section 2.3.

### 2.7 Data Analysis

The data points were analyzed with SAS Studio (SAS Institute, Cary, NC, USA) with the PROC GLM procedure. The effect of the different formulations on noodle quality and composition parameters was investigated with a two‐way analysis of variance (ANOVA) with main effects of formulation and replicate. For the investigation on flour blend composition, the five treatments comprised the blends with 0%, 5%, 15%, 25%, and 100% spent grain. For cooking quality and composition of raw and cooked noodles, the five treatments consisted of the four flour blends (0%, 5%, 15%, and 25% spent grain) and the BW noodle sample. Each batch represented one replication, using a total of three replications for each formulation and test. Mean separation was performed with Fisher's least significant difference (LSD) with 0.05 as the significance level.

## 3 Results and Discussion

Three flour blends made with spent grain and wheat flour were tested to evaluate the impact of different levels of spent grain on noodle quality; the blends contained 5%, 15%, and 25% (w/w) spent grain, and a control was formulated without the addition of spent grain. The variation was well captured by the main effects of formulation and replication, but only formulation was statistically significant, with *R* ^2^ values ranging from 0.94 to 0.99 for all the parameters.

### 3.1 Flour Protein and Ash

The compositional profiles of the wheat flour, spent grain, and the three blends were assessed (Table 2). Protein and ash contents of the wheat flour, spent grain flour, and the three blends were determined on a dry weight basis.

**TABLE 2:** Protein and ash contents of flour and flour blends.

Sample	Protein	Ash
Control	12.46 ± 0.18d	0.44 ± 0.00e
5	12.66 ± 0.13d	0.52 ± 0.00d
15	13.69 ± 0.13c	0.73 ± 0.00c
25	14.34 ± 0.24b	0.92 ± 0.00b
100	19.68 ± 0.18a	2.39 ± 0.00a
**LSD**	0.29	0.03

*Note*: Values express mean ± standard deviation (g/100 g of dry weight, *n* = 3). Means followed by the same letter within a column are not significantly different by the least significant difference method (*p* < 0.05).

Abbreviation: LSD, least significant difference.

The high protein content of spent grain offers a nutritional advantage in fortified foods (Lynch et al. 2016). The spent grain tested had 19.7% protein, higher than the 12.5% protein content of the wheat flour used in this study. As expected, the protein content of the blends increased as the proportion of the protein‐rich spent grain increased. The flour blend with 25% spent grain had 15% more protein than the control flour. The 19.7% protein content of spent grain observed in this study was slightly higher than the 18.6% average protein reported by Robertson et al. (2010) in a study of the composition of spent grain, but lower than the 31% reported by Santos et al. (2003) in a similar study. In the present study, the protein content of the developed noodles was not quantified; however, substitution of wheat flour with spent grain, which has a higher protein content, should result in spent grain noodles with a higher protein content than the control noodles made without spent grain.

Ash represents the mineral content of an ingredient (Santos et al. 2003). Minerals present in spent grain include calcium, phosphorus, iron, copper, zinc, and magnesium (Huige 2006). The 2.4% ash content of spent grain observed in this study was the same ash content previously reported by Kanauchi et al. (2001) in a study on the use of a germinated barley ingredient made from spent grain to treat ulcerative colitis. The ash levels in the three flour blends ranged from 0.52% (5% spent grain blend) to 0.92% (25% spent grain blend). Like the protein content, the ash levels also increased proportionally with increasing spent grain levels, evidenced by the higher ash percentage in the flour blends in comparison to the wheat flour (0.44% ash). A high concentration of ash is present in the bran of cereal grains. In wheat flour milling, most of the bran is removed. Because spent grain is the residue remaining after the mashing of the barley malt (Ahuja et al. 2024), the increase in ash content along with the increase in spent grain in the flour blends was expected.

Previous research reported a wide range of protein and ash in barley spent grain (Kanauchi et al. 2001; Santos et al. 2003; Mussatto and Roberto 2005; Jin et al. 2022). The compositional profile of spent grain is affected by the cereal variety used, the growing environment, and the brewing conditions (Lynch et al. 2016), therefore, differences between the proximate composition of spent grain observed in this study and in previous studies are likely attributable to differences in raw materials, processing conditions, and methods of analysis.

### 3.2 TAXs and β‐Glucans

The quantification of TAXs showed that 100 g of spent grain contained almost 6 g more TAX than a sample of 100 g of wheat flour (Table 3). The flour blends with 15% and 25% spent grain had significantly higher concentrations of TAX than the 0% and 5% spent grain blends. The levels of TAX observed for the 25% spent grain samples for flour blends, raw noodles, and cooked noodles were significantly higher than those quantified for the remaining blends, as well as for the control and commercial BW samples.

**TABLE 3:** Total arabinoxylan and β‐glucan contents of flour blends, raw noodles, and cooked noodles.

	Total arabinoxylans			β‐Glucans		
Sample	Flour blend	Raw noodles	Cooked noodles	Flour blend	Raw noodles	Cooked noodles
Control	3.92d	0.97d	1.61cd	0.23e	0.22d	0.21d
5	4.09cd	1.46c	2.07c	0.30d	0.28c	0.29c
15	4.60c	1.93b	2.71b	0.47c	0.39b	0.41b
25	5.29b	2.87a	3.50a	0.56b	0.50a	0.54a
100	9.79a	—	—	1.39a	—	—
BW	—	0.79d	1.54d	—	0.18e	0.18d
**LSD**	0.57	0.30	0.50	0.02	0.03	0.05

*Note*: Values express mean (g/100 g of dry weight, *n* = 3). Means followed by the same letter within a column are not significantly different by the least significant difference method (*p* < 0.05).

Abbreviations: BW, commercial buckwheat noodles; LSD, least significant difference.

A similar trend was observed for the β‐glucan content. Every inclusion level of spent grain resulted in a significant increase in β‐glucan content of flour blends, raw noodles, and cooked noodles. Even at low levels of spent grain inclusion, the amount of dietary fiber in the form of arabinoxylans and β‐glucans was higher in the spent grain noodles than the amount measured in the commercial BW noodles.

For all the tested blends and samples (flour, raw noodles, and cooked noodles), TAX content was higher than the β‐glucan content. In addition to the barley seed coat fractions, spent grain may contain empty aleurone cells (Mussatto et al. 2006); these grain structures contain significant amounts of arabinoxylans but low levels of β‐glucans (McNeil et al. 1975; Bacic and Stone 1981; Izydorczyk and Dexter 2008). The 1.4% β‐glucan content in the spent grain tested agrees with that previously reported in Steiner et al. (2015), who found up to 2% β‐glucan content in spent grain. A comparison of the β‐glucan content of wheat flour and spent grain flour showed 2.5 times more β‐glucans in spent grain than in the tested wheat flour, highlighting the nutritional benefit of spent grain as an ingredient.

For all the inclusion levels tested, the TAX content decreased from flour to raw noodles and slightly increased from raw to cooked noodles. In a previous study, different TAX contents in wheat flour, batter, and pancakes were also observed (Kiszonas et al. 2015). Arabinoxylan molecules can be degraded in the presence of strong acids or enzymes (Kiszonas et al. 2015); however, such conditions are not present in noodle processing. Therefore, it is likely that some of the arabinoxylan molecules were inaccessible for extraction and quantification in the raw noodle matrix but became available through the cooking process. The β‐glucans levels were consistent across flour blends, raw noodles, and cooked noodles.

Based on the analysis conducted, the consumption of 100 g of cooked 25% spent grain noodles would provide an average of 2.2 g more dietary fiber—in the form of arabinoxylans and β‐glucans—than would be provided by the consumption of 100 g of cooked noodles without spent grain or the same portion of commercial BW noodles. Considering the 140 g reference amount customarily consumed per eating occasion of prepared noodle stated by the Code of Federal Regulation (21 C.F.R. § 101.12) (Food and Drug Administration [FDA] 2018), the consumption of 140 g of control noodles made with wheat flour would provide 2.5 g of dietary fiber in the form of arabinoxylans and β‐glucans; ingesting 140 g of cooked 25% spent grain noodles would provide 5.7 g of combined arabinoxylans and β‐glucans, equivalent to 20.4% of the 28 g of dietary fiber recommended by the United States Code of Federal Regulations as the Daily Reference Value (DRV) for adults (21 C.F.R. § 101.9) (FDA 2023). In this study, other dietary fiber fractions such as lignin and cellulose were not quantified, and the composition is reported on a dry weight basis, meaning that the quantities of arabinoxylans and β‐glucans would be slightly higher on an “as is” basis that truly represents how the product is consumed. Higher levels of dietary fiber per serving (7.2–8.5 g) were achieved by Izydorczyk et al. (2005) in their study on the enrichment of Asian noodles. However, Izydorczyk and coworkers used high amylose and waxy barley grains, naturally higher in β‐glucans than the normal‐starch barley used in this study, to produce their fiber‐rich ingredients.

### 3.3 Noodle Quality

The prepared noodle samples were tested for quality parameters (Table 4). The inclusion of 5% spent grain did not significantly affect the cooking time of the noodles, and no significant difference (*p* < 0.05) was observed in the cooking time of noodle samples with 0% and 5% spent grain, and the commercial BW noodle sample. Noodles made with higher levels of spent grain cooked faster than those made with wheat flour and commercial BW noodles, and increasing levels of spent grain led to noodles cooking faster. On average, noodles made with 25% spent grain were fully cooked 2 min 10 s faster than noodles without spent grain. In a study on the nutritional enrichment of Asian noodles, Izydorczyk et al. (2005) also observed a significant reduction in cooking time when including 25% of a fiber‐rich ingredient made from hull‐less barley. Noodles are fully cooked when the starch absorbs water and fully gelatinizes. During the mashing step of the brewing process, most of the water‐soluble components are removed with the liquid fraction (Steiner et al. 2015). The resulting insoluble waste residue is the spent grain, rich in fiber and protein (Ahuja et al. 2024). Typically, 30%–50% of spent grain is composed of non‐starch polysaccharides (Lynch et al. 2016); the amount of starch in spent grain is negligible (Shi et al. 2023). The minimal amount of starch in spent grain and, consequently, in the noodles may shorten the cooking time of the developed product. The shorter cooking time also could be minimally attributed to the higher dough hydration levels (Table 1) used for increasing levels of spent grain. Noodles are valued as practical and convenient products (Meenu et al. 2023). Therefore, the shorter cooking time for the spent grain noodles stands as an advantage of the developed product.

**TABLE 4:** Quality parameters for noodles with spent grain and commercial buckwheat noodles.

Sample	Cooking time (min:s)	Weight increase (%)	Cooking loss (%)	Firmness (g)
Control	5:40 ± 00:17a	127.0 ± 1.1b	14.4 ± 0.5a	174.1 ± 4.6c
5	5:30 ± 00:00a	121.5 ± 1.8c	13.2 ± 0.2b	178.3 ± 7.4c
15	4:10 ± 00:17b	114.7 ± 5.0d	10.6 ± 0.4c	201.5 ± 7.1b
25	3:30 ± 00:00c	109.2 ± 0.8e	10.0 ± 0.1cd	201.7 ± 10.3b
BW	5:20 ± 00:17a	159.2 ± 0.7a	9.7 ± 0.1d	244.8 ± 4.1a
**LSD**	00:27	4.8	0.6	6.5

*Note*: Values express mean ± standard deviation (*n* = 3 for all parameters except *n* = 9 for firmness). Means followed by the same letter within a column are not significantly different by the least significant difference method (*p* < 0.05).

Abbreviations: BW, commercial buckwheat noodles; LSD, least significant difference.

*Noodle weight increase*, also referred to in the literature as *cooking yield* or *water absorption*, represents the water absorbed by noodle strands during cooking. Weight increase values above 100% are indicative of good noodle quality (Hatcher 2010); all the tested noodles were above this threshold (Table 4). The commercial noodles made from BW and wheat flour showed a 159% weight increase, within the 146%–163% range reported by X. N. Guo et al. (2017) for noodles made with BW and wheat flour. The tested commercial noodles absorbed more water than the samples developed with spent grain. Increasing the level of spent grain added to the formulation resulted in a decrease in the total water uptake by the noodles. Noodles made with 25% spent grain showed the lowest weight increase (109%) among the tested samples. Noodle formulated with spent grain required up to 3% more water to prepare the dough (Table 1), and this higher dough hydration could have inhibited the absorption of more water by the noodle strands during cooking, influencing in the lower weight increase observed for spent grain noodles. Spent grain has a high capacity for water absorption (Huige 2006), so the low water absorption by these noodles with higher levels of spent grain was unexpected. However, the substitution of starch‐rich wheat flour with an ingredient containing protein and insoluble fiber and insignificant levels of starch—such as spent grain—is likely to have caused the lower weight increase of the developed noodles. Consumers and industry recognize weight increase as a proxy for noodle quality (Niu and Hou 2019). High weight increase is generally desirable (G. Guo et al. 2003; Niu and Hou 2019), but very high weight increase can lead to undesirable degrees of softness and stickiness (Lee et al. 2005; Soncin Alfaro et al. 2023).

*Noodle cooking loss*, the loss of noodle particles into cooking water, indicates noodle integrity (Niu and Hou 2019). Cooking loss was lower for noodles made with higher levels of spent grain than for noodles made only with wheat flour (Table 4). Spent grain is rich in cellulose, non‐cellulosic polysaccharides, and lignin (a polyphenolic macromolecule) (Mussatto et al. 2006). The considerable amount of fiber and protein added to the noodles by the inclusion of spent grain may have created a matrix that strongly entrapped the components, leading to fewer solids leaching into the water. In addition, the shorter cooking times for noodles with spent grain could also lower cooking loss. Noodles without spent grain were in boiling water for longer, therefore allowing for more solids to leach.

*Noodle texture* significantly influences a consumer's willingness to repeat the purchase (Hatcher 2010), hence the critical importance of assessing noodle firmness. Noodles made with 15% and 25% spent grain showed the highest firmness among the produced samples (Table 4). Given the low levels of weight increase observed for these samples, a firmer texture for noodles with 15% and 25% spent grain was expected. Higher levels of protein result in harder noodles (Crosbie and Ross 2004), hence the higher protein content of spent grain also contributed to the higher noodle firmness.

The commercial BW noodles were firmer than all the lab‐sheeted produced samples (Table 4). Changes in the textural characteristics were anticipated for noodles made with different ingredients under different processing conditions. The ingredients used in the commercial noodles were different from the ingredients used in the noodles developed in this study, and no details are known about the processing steps that led to the quality parameters observed for the BW noodles. Given that the BW noodles were firmer than those made with spent grain, and the tested BW noodles are commercially available, the firmness of the spent grain noodles is not expected to lead to consumer rejection. However, consumer acceptance of texture was not investigated in this study. Measuring texture with the use of instruments allows for a cost‐effective way to evaluate how changes in noodle formulation can impact noodle texture (Ross 2020). Further studies should include a sensory panel to evaluate the textural properties of the developed noodles by consumers.

**FIGURE 1:**
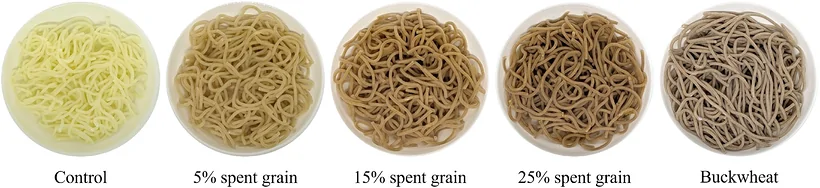
Noodles made from wheat flour (control), blends of wheat flour and spent grain (5%, 15%, and 25% spent grain, w/w), and commercial buckwheat noodles.

The Maillard reactions during kilning are responsible for malt color development (Palmer 2017). Additional color development likely occurred during drying of spent grain. Spent grain flour is darker than refined wheat flour, evidenced by the lower 67.7 L* value observed for the 100% spent grain flour compared to the 91.5 L* value for the wheat flour (control) (Table 5, Figure 1). As expected, the inclusion of spent grain in the formulation of noodles darkened flour blends, raw noodles, and cooked noodles (decreased L* value). Spent grain also showed high levels of redness (+a*) and yellowness (+b*); therefore, noodles with spent grain were also redder and more yellow. The brown color of spent grain can restrict its use in replacing commonly used flours (Mussatto et al. 2006). Like results observed in this study, a darkening effect was observed by Ktenioudaki et al. (2013) in their study of baked snacks with spent grain inclusion. Those snacks with 25% spent grain had lower acceptability as evaluated by a sensory panel with 20 individuals (Ktenioudaki et al. 2013). Even though the color and the aroma of the baked snacks were also evaluated, taste and texture were the attributes that most influenced the acceptance of the spent grain snacks by the panelists (Ktenioudaki et al. 2013).

**TABLE 5:** Color assessment for flour blends, raw noodles, and cooked noodles.

	Flour blend			Raw noodles			Cooked noodles		
Sample	L*	a*	b*	L*	a*	b*	L*	a*	b*
Control	91.5a	2.5e	11.7d	81.2a	0.3e	18.5c	56.8a	−2.6e	6.7e
5	87.3b	3.1d	11.3e	67.5b	5.4d	19.0ab	50.3b	1.6d	12.4c
15	82.5c	3.8c	12.3c	58.0c	6.6b	18.8bc	45.6d	6.2c	14.6a
25	79.6d	4.2b	13.1b	55.3d	6.7a	19.4a	42.5e	7.7a	14.1b
100	67.7e	5.5a	18.0a	—	—	—	—	—	—
BW	—	—	—	55.8d	6.1c	12.6d	48.0c	6.4b	9.5d

*Note*: Values express mean (*n* = 3 for flour blend and *n* = 9 for noodles). Means followed by the same letter within a column are not significantly different by the least significant difference method (*p* < 0.05).

Abbreviation: BW, commercial buckwheat noodles.

The evaluation of a commercial sample of BW noodles, prompted by the visual similarities with spent grain noodles, aimed at investigating if spent grain noodles could be an alternative to BW for individuals who cannot consume BW, with the advantage of higher levels of protein and fiber measured as TAXs and β‐glucans. The assessment of the color of noodles with 25% spent grain and commercial BW noodles showed that all the color parameters were significantly different (*p* < 0.05) for raw and cooked noodles, except for the L* value for raw noodles. The L* (lightness) and a* values (red to green color) observed for cooked BW were between the values observed for the noodles with different percentages of spent grain.

The use of colorimeters is an instrumental approach to assess noodle color and evaluate the product appearance (Ross 2020). However, because noodle color directly influences consumer acceptability (Niu and Hou 2019; Ross 2020), further investigation with a sensory panel could assess if a difference in the color of the tested noodles would be perceived and/or accepted by consumers. A comparison between attributes of spent grain noodles and whole wheat noodles could also be conducted. Even though consumers from different generations reported willingness to purchase upcycled foods (Zhang et al. 2021), the confirmation or denial of acceptance of the developed spent grain noodles would, ideally, require a more robust assessment by a consumer sensory panel. Consumer acceptance of the developed noodles was not yet investigated and represents a limitation of this study. Notably, future studies should also investigate the sensory evaluation of the appearance and flavor of spent grain.

## 4 Conclusion

This study showed that the inclusion of spent grain in yellow alkaline noodles increased the dietary fiber content of a developed product in the form of TAXs and β‐glucans while keeping desired quality characteristics. Noodles with spent grain cooked faster than control noodles made with wheat flour. Spent grain noodles had lower cooking loss, and higher levels of TAX and β‐glucans than noodles made without spent grain. The firmness of noodles with 15% and 25% spent grain, measured using a texture analyzer, was slightly higher than the firmness of noodles with 0% and 5% spent grain, but lower than the firmness of a commercial BW noodle sample. Spent grain noodles visually resemble BW noodles, with the added benefit of cooking faster and containing more dietary fiber in the form of TAX and β‐glucans.

This study adds to the research body by demonstrating that spent grain, a byproduct of the brewing industry, can be successfully incorporated into yellow alkaline noodles to take advantage of the nutritional composition of spent grain and to reduce waste. Future studies could further investigate consumer acceptance of the appearance, taste, and texture of spent grain noodles, as well as whether spent grain made from other barley malt varieties or a combination of cereals would result in different noodle quality characteristics.

## Author Contributions

**Gabriely M. Soncin Alfaro**: conceptualization, investigation, methodology, formal analysis, data curation, writing – original draft, visualization. **Shaun J. Clare**: conceptualization, investigation, methodology, writing – review and editing. **Robert S. Brueggeman**: resources, funding acquisition, supervision. **Sean M. Finnie**: conceptualization, resources, funding acquisition, writing – review and editing. **Alecia M. Kiszonas**: conceptualization, resources, funding acquisition, supervision, writing – review and editing.

## Conflicts of Interest

The authors declare no conflicts of interest.

## Data Availability

The data that supports the findings of this study are available from the corresponding author upon reasonable request.
